# A Prospective Study on Correlation of First Trimester Crown-Rump Length With Birth Weight

**DOI:** 10.7759/cureus.28781

**Published:** 2022-09-04

**Authors:** Shweta Patel, Aditya Sarkar, K Pushpalatha

**Affiliations:** 1 Obstetrics and Gynaecology, All India Institute of Medical Sciences, Bhopal, IND

**Keywords:** ultrasound, birth weight, ultrasonography, low birth-weight, crown-rump length, first trimester scan

## Abstract

Background

Ultrasound examination has been a crucial part of the evaluation of fetal health during pregnancy. It has become more accurate over the past few decades as a result of advances in radiodiagnostic imaging. While obstetric ultrasonography in the first trimester has been utilized extensively for gestational age assessment and confirmation of fetal viability, the imaging technique has seen little exposure in predicting pregnancy outcomes. This study was thus undertaken to find out any possible association between one of the first trimester parameters, i.e. crown-rump length (CRL) noted at the beginning of a pregnancy, and the birth weight of the neonate.

Methods

This prospective cohort study conducted at a teaching hospital in India spanning over a period of eighteen months included women with a spontaneously conceived intrauterine pregnancy at six to ten weeks period of gestation as calculated from the last menstrual period. Transvaginal sonography was performed for all such women and the CRL was noted. These CRL values were then compared to a standard nomogram and assigned to either of three categories i.e. CRL <5^th^ centile, 5^th ^to 95^th^ centile, or >95^th^ centile. The women were then followed up at the hospital with standard care till the end of their pregnancy, and finally, the birth weights were noted. Data were recorded in an MS Excel spreadsheet program and analysis was performed with regard to CRL in the first trimester and birth weights using SPSS v23 (IBM Corp.).

Results

Crown-rump lengths and birth weights of 104 cases were evaluated. The mean age of the study population was 26.6 years and the mean period of gestation (weeks) was 8.28 ± 1.01. The incidence of low birth weight (LBW) in the study was 22.1%. The distribution of LBW was significantly different between the three CRL categories (χ2 = 15.868, p = <0.001), being considerably higher in the CRL <5^th^ centile category. No embryos with CRL >95^th^ centile had low birth weight.

Conclusions

Our study suggested a congruence between the crown-rump length of an embryo noted in the first trimester and its weight at birth, with low birth weight being a fairly common occurrence in the deficient CRL category. This study highlights the role of a carefully performed first-trimester ultrasound examination in possibly predicting an adverse pregnancy outcome such as low birth weight and the probable inherent tendency of growth restriction in fetuses that are destined to develop the same.

## Introduction

Diagnostic ultrasonography utilizes high-frequency sound beams to produce images [1]. Nowadays, ultrasound evaluation has become a pivotal part of obstetric examination and assessment of the well-being of the fetus. In the past few decades, ultrasound examination in the first trimester has been utilized for confirming fetus viability, estimating gestational age, and also for ruling out life-threatening complications such as ruptured ectopic pregnancy [2,3].

While the use of first-trimester parameters for gestational age assessment has been vastly popular and widely used, their role in predicting pregnancy outcomes has seen limited exposure. While some investigators have found some of the first trimester ultrasound parameters including mean sac diameter (MSD), crown-rump length (CRL), yolk sac diameter (YSD), embryonic/fetal heart rate (FHR), and subchorionic hemorrhage to be possible predictors of adverse pregnancy outcomes, others have dismissed the significance of such associations [4,5,6,7]. This study attempts to find out the correlation between the crown-rump lengths (CRL) observed in the first-trimester ultrasound and the birth weight of the neonate upon completion of the pregnancy, with low birth weight (absolute weight of <2500 g regardless of gestational age) being a significant contributor to neonatal morbidity and mortality, especially in the South Asian population.

## Materials and methods

Study design

This was a prospective cohort analytical study conducted over a period of 18 months at a government teaching hospital in Central India.

Inclusion criteria

Women with previously regular menstrual cycles who conceived spontaneously, with a period of gestation between 6 weeks to 10 weeks (calculated from LMP) were included in the study and subjected to history-taking, general physical, obstetric examination, and transvaginal sonography (TVS).

Exclusion criteria

Based on the history and ultrasound evaluation, patients with gravida≥4, multifetal pregnancy, ectopic and molar pregnancies, congenital uterine anomalies, and those with a history of recurrent pregnancy loss (RPL), ovulation induction/assisted reproductive technology conception, and significant past medical, surgical, or poor obstetric history were excluded from the study.

Data collection

CRL was measured in the sagittal plane as per standard protocol and precautions in units of centimeters, and percentiles were calculated based on the nomogram developed by Papaioannou et al [8]. Individual CRL values were then allotted to three categories: CRL <5th centile, 5th to 95th centile, or >95th centile based on the aforementioned nomogram. These women received follow-up in every subsequent visit until the end of their pregnancy, and a routine physical examination including a general survey and obstetric examination was done for all patients. Finally, birth weights were noted at parturition.

Statistical analysis

Data were coded and recorded in the Microsoft Excel spreadsheet program. Data analysis was performed using SPSS v23 (IBM Corp.).

## Results

A total of 120 patients were included in the study. The mean period of gestation (weeks) was 8.28 ± 1.01, while the mean age of the study population was 26.6 years. Approximately 56% of the patients in the study were nulliparous, while 44% were multiparous. The fetal pole was not visualized in seven patients at the time of TVS. Of the remaining 113 patients, 9 underwent spontaneous pregnancy losses. Thus, the birth weight results presented here are representative of 104 patients who underwent a live birth.

The variable birth weight (g) was not normally distributed in the three subgroups of the variable CRL Percentile (Table 1). Thus, non-parametric tests (the Kruskal Wallis Test) were used to make group comparisons (χ2 = 12.067, p = 0.002). The mean birth weights in the <5th, 5th-95th, and >95th centile categories were 2457, 2823, and 3131 g, respectively, with a strength of association (Kendall’s Tau) of 0.28 (i.e., small effect size).

**Table 1:** Comparison of the 3 Subgroups of the Variable CRL Percentile in Terms of Birth Weight (g) (n = 104)

Birth Weight (g)	CRL Percentile			Kruskal Wallis Test	
	<5th	5th-95th	>95th	χ2	p value
Mean (SD)	2457.47 (523.68)	2822.78 (498.20)	3131.25 (337.45)	12.067	0.002
Median (IQR)	2421 (2175-2700)	2900 (2600-3065)	3250 (2912.5-3345)		
Range	1640 - 3520	585 - 3900	2540 - 3480		

**Figure 1:**
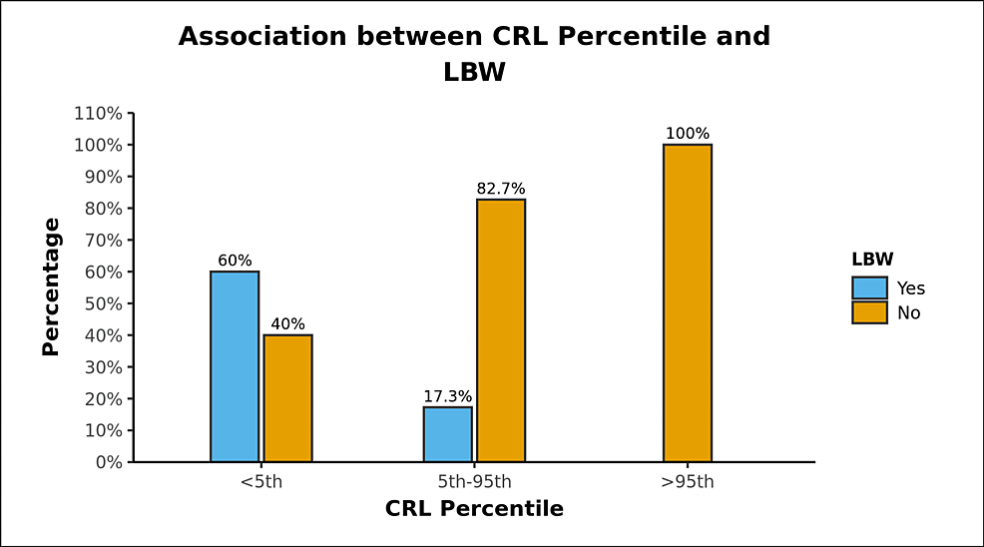
Incidence of Low Birth Weight (LBW) in different centile categories

Approximately 22.1% (23 out of 104) of women gave birth to a neonate with LBW. A significant difference between the various groups in terms of distribution of LBW (χ2 = 15.868, p = <0.001) was noted, with a strength of association between the two variables (Bias Corrected Cramer’s V) being 0.37 (i.e., moderate association) (Table 2 and Figure 1).

**Table 2:** Association Between CRL Percentile and LBW (n = 104)

LBW	CRL Percentile				Fisher's Exact Test	
	<5th	5th-95th	>95th	Total	χ2	P Value
Yes	9 (60.0%)	14 (17.3%)	0 (0.0%)	23 (22.1%)	15.868	<0.001
No	6 (40.0%)	67 (82.7%)	8 (100.0%)	81 (77.9%)		
Total	15 (100.0%)	81 (100.0%)	8 (100.0%)	104 (100.0%)		

## Discussion

In our study, LBW was found to be recurring in the CRL <5th centile category, with the incidence being as high as 60%, compared to 17% in the 5th-95th centile CRL category (p-value <0.001). Similar to our study, Leung et al. analyzed 2760 cases and found fetal CRL expressed as their standardized z-scores (Z-CRL) to be independent predictors of birth weight [9]. Carbone et al. found Z-CRL<−1 SD could be used as a screening tool for small for gestational age [10]. Similarly, Park et al. found smaller than expected crown-rump lengths to be predictors of low birth weight, and the highest power to predict LBW was found at a CRL cut-off of 26.5 mm [11]. Meanwhile, Halscott et al. found that first trimester ultrasound markers including CRL did not have a high enough positive predictive value for predicting fetal growth restriction amongst other obstetric complications [12]. None of the embryos/fetuses with CRL>95th centile had LBW. The mean birth weights were found to be congruent with their CRLs earlier in the pregnancy, being 2457, 2823, and 3131 g in the CRL<5th, 5th-95th, and >95th centile categories, respectively. These findings suggest an inherent tendency of inhibited or restricted growth in fetuses with CRLs that are smaller than expected for their gestational age. Such findings could allow radiologists and obstetricians to possibly identify women likely to give birth to neonates with LBW. Therefore, in the South Asian population, where the prevalence of LBW is among the highest in the world, the first-trimester ultrasound could prove to be a promising screening tool. The majority of management choices for such patients would center on increased monitoring and counseling, in addition to making sure the mother is getting enough required nutrients.

Limitations

The study was undertaken during the COVID-19 pandemic, which resulted in a small sample size since a large number of patients who were initially included in the study were lost to follow-up. The volume of subchorionic hemorrhage and the yolk sac morphology were not accounted for in this study. All women with gravida ≥4 and multifetal pregnancy were also excluded from the study to eliminate as many confounding factors as possible from the study.

## Conclusions

This study demonstrates an associative correlation between CRL in the first trimester with birth weights at the end of pregnancy, with the risk of LBW being appreciably higher in fetuses with small CRLs. Thus, a meticulously measured CRL could give valuable insights in predicting the growth potential and hence birth weights of neonates. This could offer a better perspective to the obstetrician for increased vigilance, and to the expecting parents for better preparedness. However, this path should be trodden carefully and only after more extensive and elaborate studies, heeding the vast plethora of factors that could possibly impact the birth weight of a neonate. A larger multi-centric study with due consideration to other factors impacting pregnancy outcomes could help corroborate the findings of this study and the utilization of CRL as an important prognostic tool.
